# Supplementary figures and images for: The HN protein of Newcastle disease virus induces cell apoptosis through the induction of lysosomal membrane permeabilization
Source: PLoS Pathog. 2024 Feb 14;20(2):e1011981. doi: 10.1371/journal.ppat.1011981 (PMC10866534; doi:10.1371/journal.ppat.1011981)

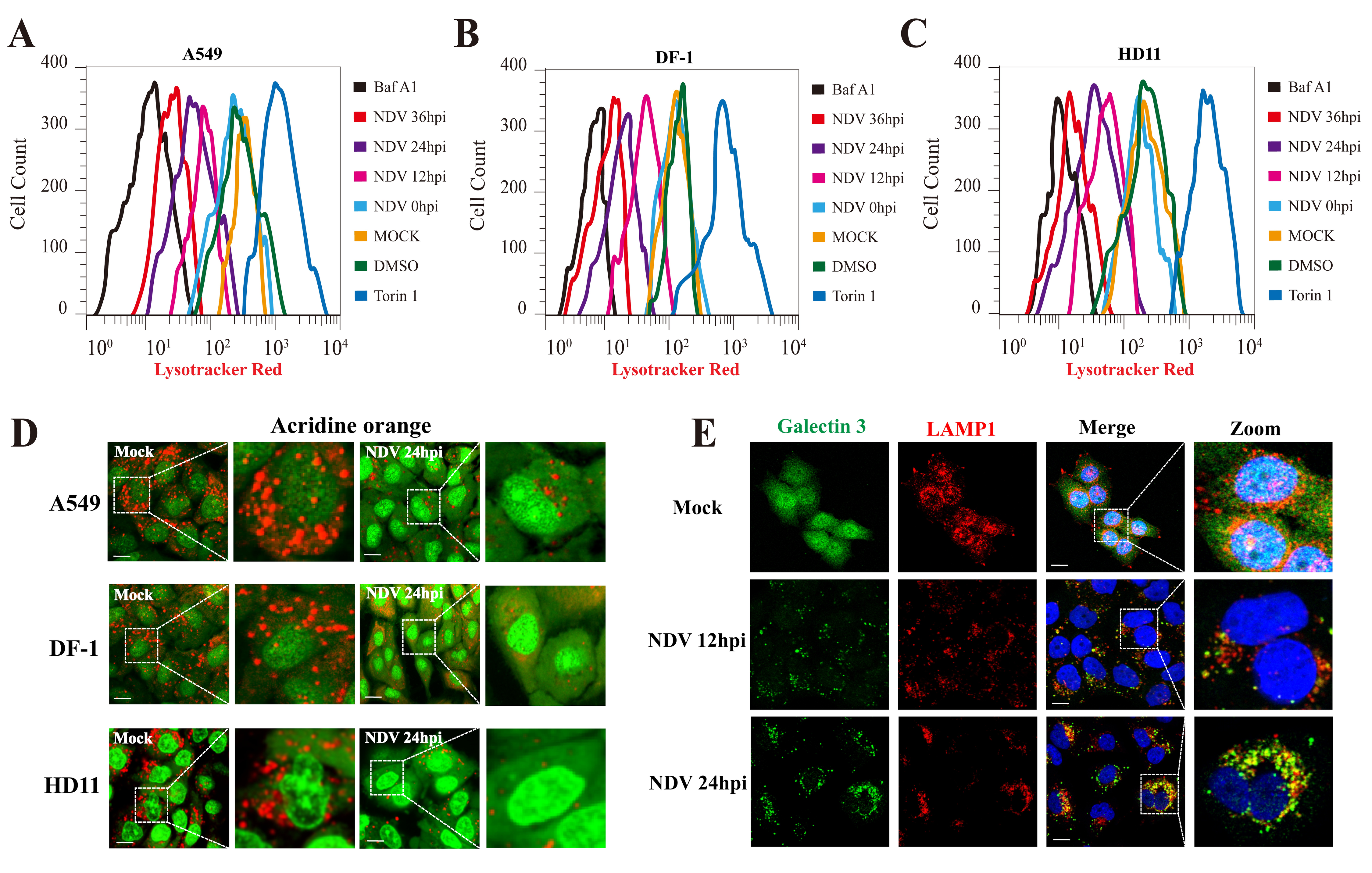

Supplement: S1 Fig — (A-C) A549 (A), DF-1 (B), and HD11 (C) cells were infected with Herts/33 at 0.01 MOI for the indicated time points. Torin 1 (1 μM, 4h) and Baf A1 (1 μM, 4h) were used as positive and negative controls, respectively. Lysotracker fluorescence signals were detected by flow cytometry after staining the cells with Lysotracker (100 nM) for 1h. (D) A549, DF-1, and HD11 cells were infected with Herts/33 at 0.01 MOI for 24h or mock-infected. Then, AO green and red fluorescence signals were detected by confocal microscopy following incubation with AO (1 μg/mL) for 30 min. Scale bars, 20 μm. (E) A549 cells were infected with Herts/33 at 0.01 MOI for the indicated time points or mock-infected. The cells were then stained with rabbit anti-galectin 3 (green) and mouse anti-LAMP1 (red) antibodies and were observed by confocal microscopy. Scale bars, 20 μm. (TIF) [file ppat.1011981.s001.tif]

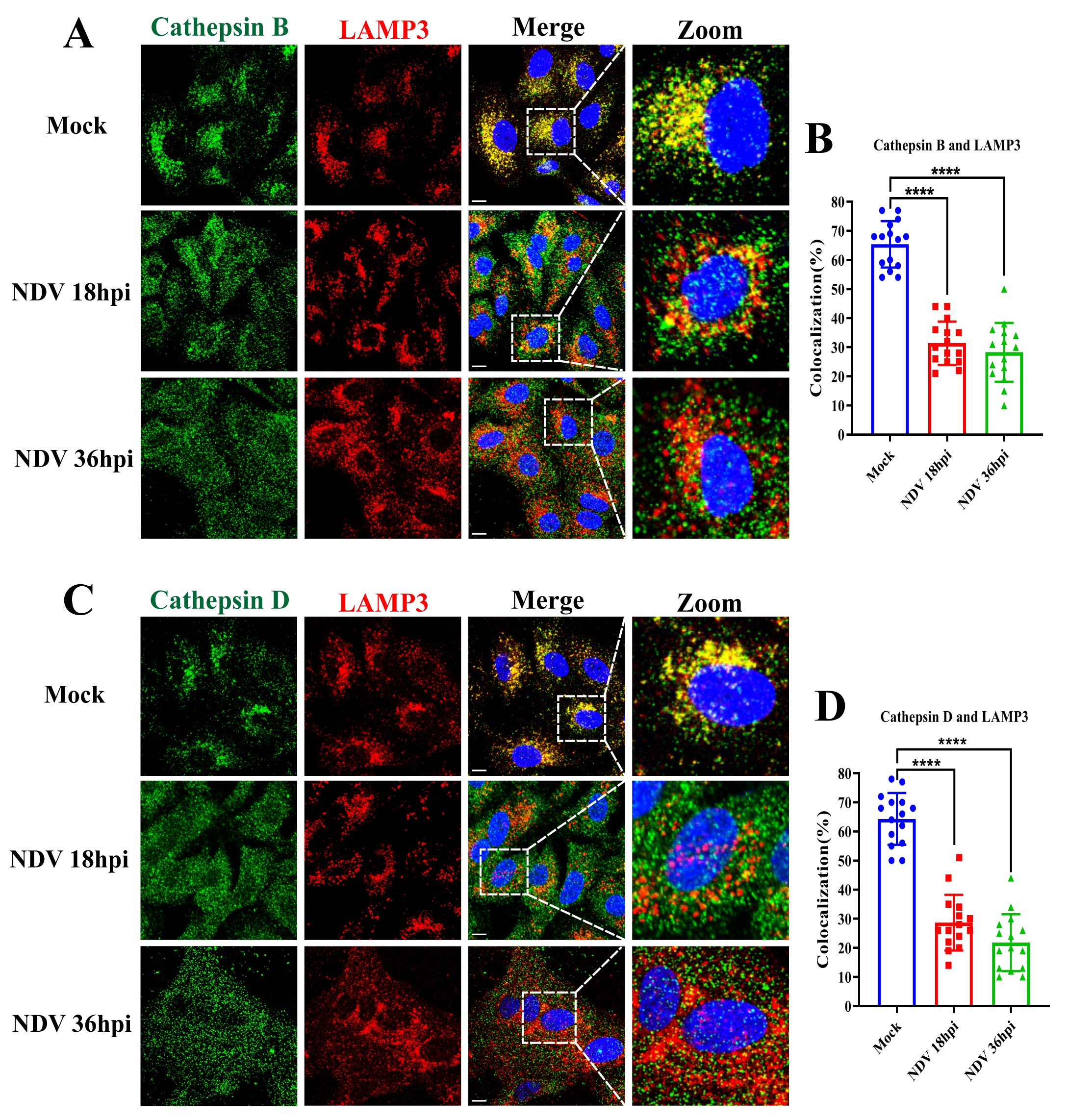

Supplement: S2 Fig — (A&C) A549 cells were infected with Herts/33 at 0.01 MOI for the indicated time points or mock-infected. The colocalization of CTSB (A) or CTSD (C) with LAMP3 was detected using confocal microscopy. Scale bars, 20 μm. (B&D) Manders’ Colocalization Coefficients of CTSB (B) or CTSD (D) with LAMP3 were quantified by ImageJ software. Error bars represent SDs for 15 cells (B&D). Significance was assessed using one-way ANOVA with Dunnett’s multiple comparison test. (TIF) [file ppat.1011981.s002.tif]

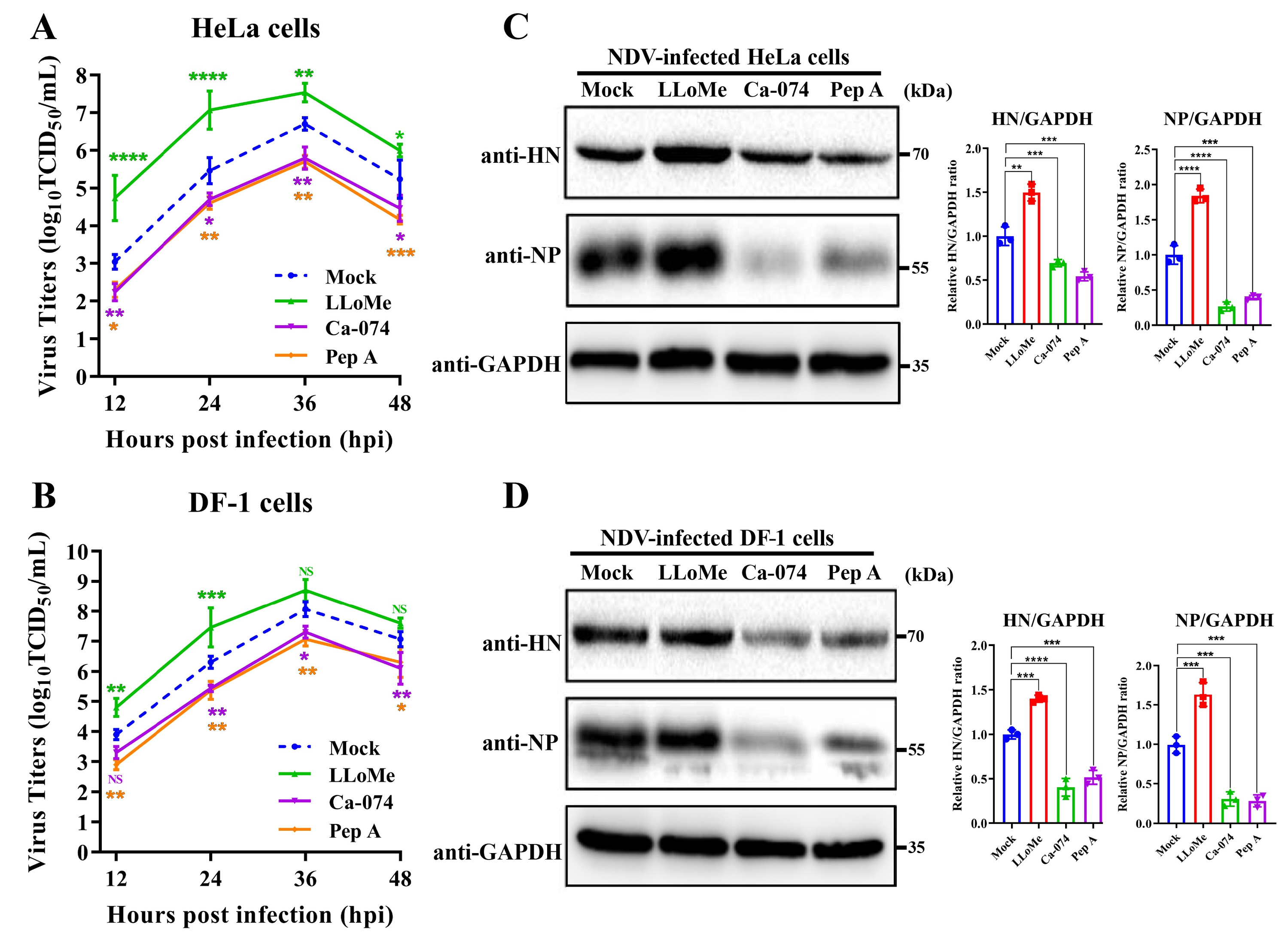

Supplement: S3 Fig — (A&B) HeLa (A) and DF-1 (B) cells were pretreated with LLoMe (500 nM), Ca-074 (5 μM), Pep A (5 μM), or mock-treated for 3h, respectively. Subsequently, the cells were infected with Herts/33 at 0.01 MOI along with the corresponding chemicals in the cell culture medium. Culture supernatants were collected at the indicated time points, and viral titers were determined by the 50% tissue culture infectious dose (TCID50) assay. (C&D) HeLa (C) and DF-1 (D) cells were pretreated and infected as described above. Protein levels of NP and HN were assessed by Western blotting after 24h of NDV infection. The relative intensity ratio of the indicated proteins, normalized to GAPDH, was quantified using ImageJ software and is presented on the left side of the panel. All error bars are SDs for a triplicate analysis of three independent experiments. Significance was assessed using Two-way ANOVA with Dunnett’s multiple comparisons test (A&B) or one-way ANOVA with Dunnett’s multiple comparison test (C&D). (TIF) [file ppat.1011981.s003.tif]

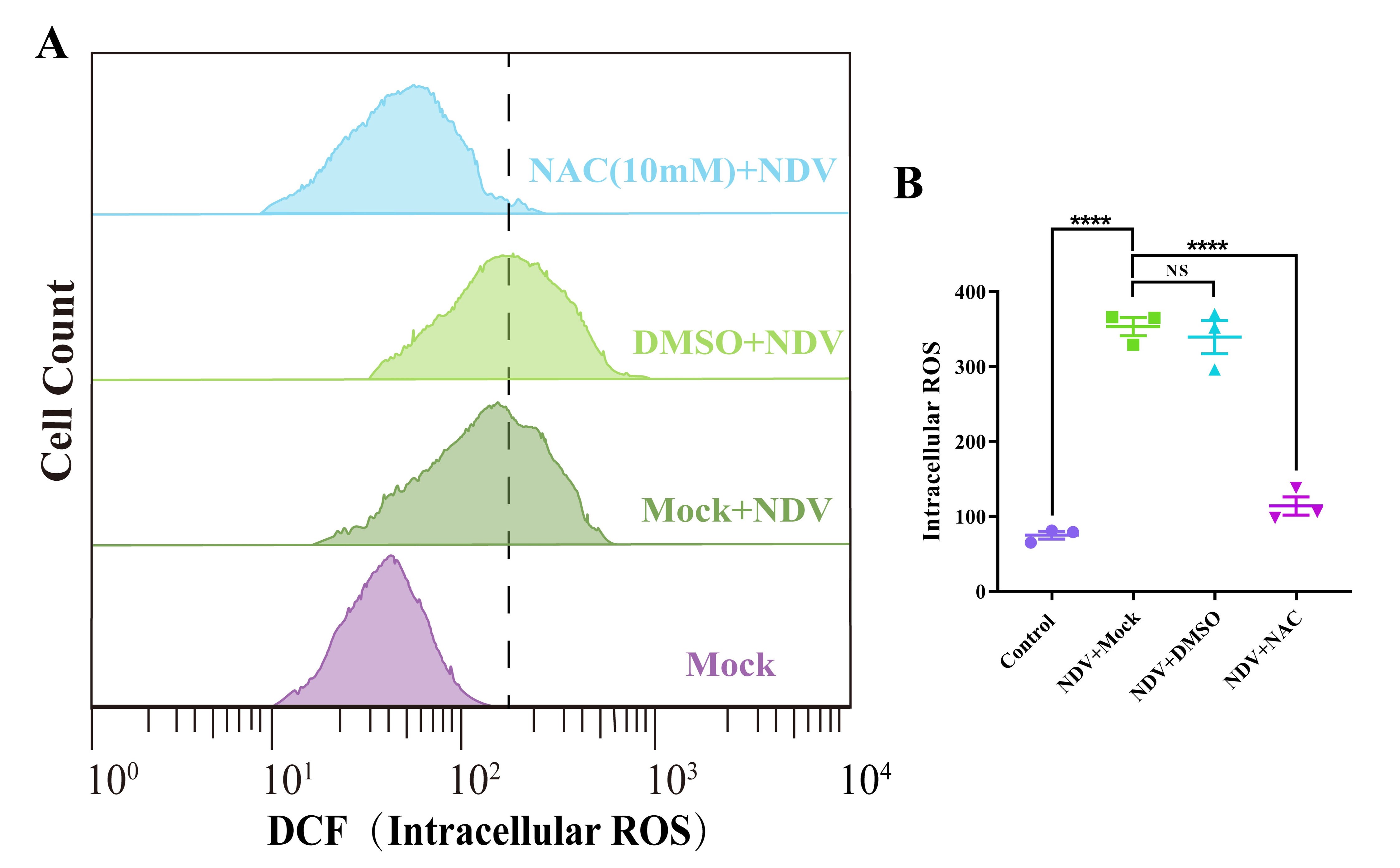

Supplement: S4 Fig — (A) HeLa cells were treated with NAC (2 mM) for 24h after absorption with Herts/33 at 0.01 MOI for 1h. Intracellular ROS levels were measured by flow cytometry using DCFH-DA staining. (B) The quantitative data are presented as means ± SD (n = 3). Significance was assessed using one-way ANOVA with Dunnett’s multiple comparison test. (TIF) [file ppat.1011981.s004.tif]

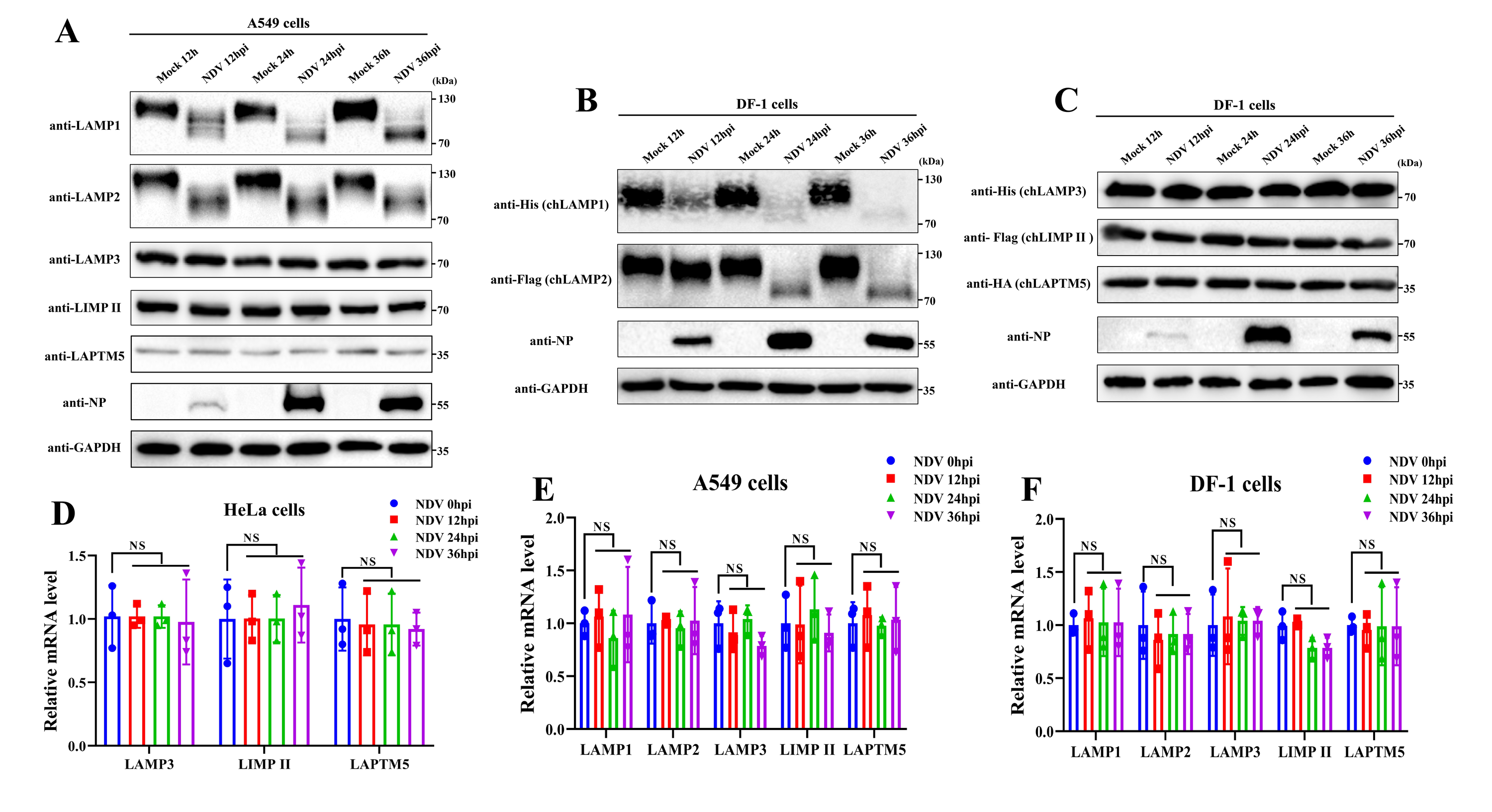

Supplement: S5 Fig — (A) A549 cells were infected with Herts/33 at 0.01 MOI or mock-infected. After the indicated time points, the protein levels of indicated lysosome membrane proteins were detected by Western blotting assay. (B&C) DF-1 cells were co-transfected with plasmids expressing avian LAMP1 (His tag) and LAMP2 (Flag tag) (B or co-transfected with plasmids expressing avian LAMP3 (His tag), LIMP II (Flag tag) and LAPTM5 (HA tag) (C). Forty-eight hours after transfection, the cells were infected with Herts/33 at 0.01 MOI or mock-infected for 24h. Protein levels of indicated proteins were then detected by Western blotting assay. (D-F) HeLa (D), A549 (E) and DF-1 (F) cells were infected with Herts/33 at 0.01 MOI or mock-infected. After the indicated time points, the mRNA levels of indicated lysosome membrane proteins were detected by qRT-PCR assay. The mRNA levels were normalized to GAPDH and calculated using the 2−ΔΔCt method. All the quantitative data are presented as means ± SD (n = 3). Significance was assessed using Two-way ANOVA with Dunnett’s multiple comparisons test. (TIF) [file ppat.1011981.s005.tif]

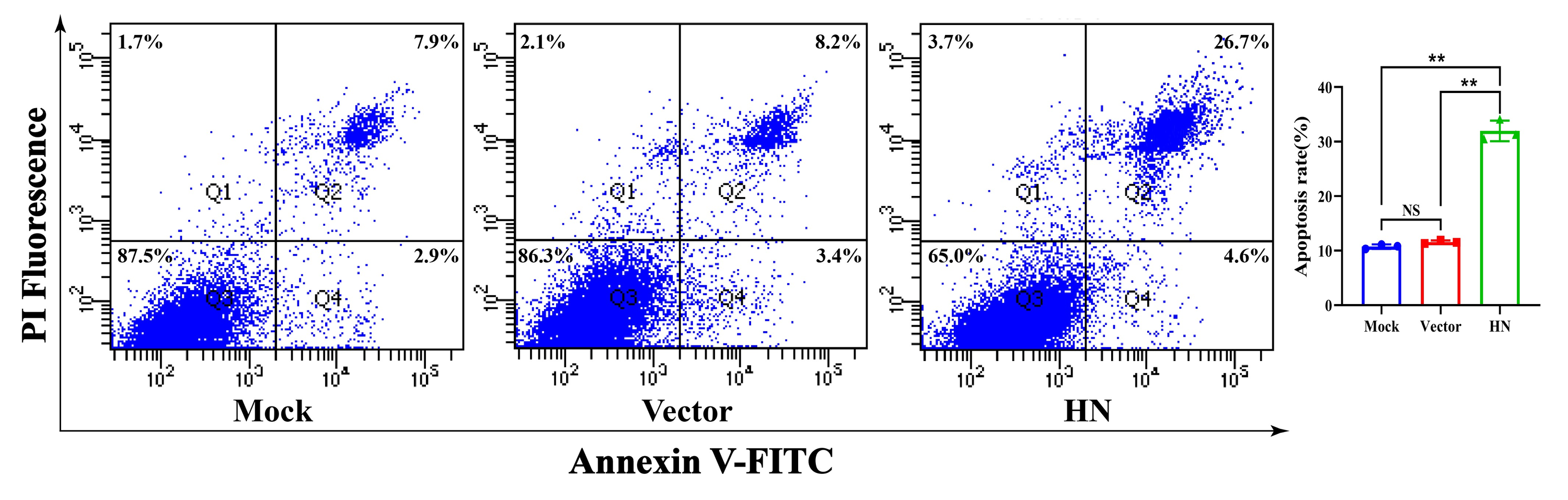

Supplement: S6 Fig — HeLa cells were transfected with a plasmid expressing HN protein (1.6 μg), or empty vector plasmid (1.6 μg), or mock-transfected. The apoptosis rate was measured by AnnexinV-FITC/PI staining using flow cytometry after transfection for 48h. Quantitation of apoptosis rate is calculated as means ± SD (n = 3) and shown in the right panel. Significance was assessed using One-way ANOVA with Dunnett’s multiple comparisons test. (TIF) [file ppat.1011981.s006.tif]

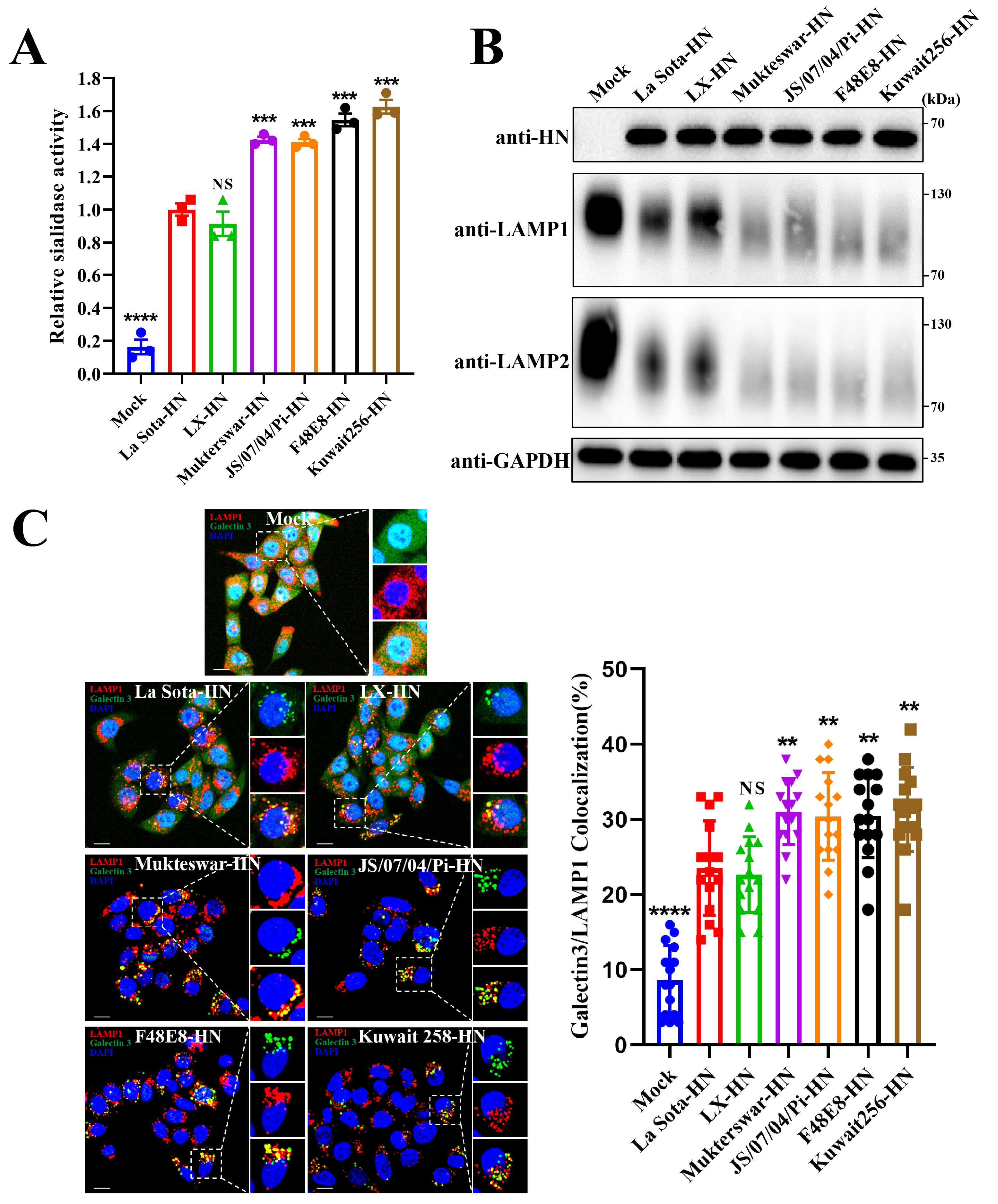

Supplement: S7 Fig — (A&B&C) HeLa cells were transfected with indicated plasmids expressing HN protein of distinct NDV strains (1.6 μg) or mock-transfected for 48h. (A) The sialidase activity was measured using the Neuraminidase Assay Kit. (B) Protein levels of indicated proteins were detected by Western blotting assay. GAPDH was used as a normalized control. (C) The LMP levels were observed by confocal microscopy using anti-galectin 3 (green) and anti-LAMP1 (red) antibodies. Scale bars, 20 μm. Manders’ Colocalization Coefficients of galectin 3 with LAMP1 were quantified by ImageJ software and shown on the right. Error bars represent SDs for triplicate analyses of three independent experiments (A), or SDs for 15 cells (C). All significance analyses were assessed using One-way ANOVA with Dunnett’s multiple comparisons test. (TIF) [file ppat.1011981.s007.tif]
