# Supplementary material for: The HN protein of Newcastle disease virus induces cell apoptosis through the induction of lysosomal membrane permeabilization
Source: PLoS Pathog. 2024 Feb 14;20(2):e1011981. doi: 10.1371/journal.ppat.1011981 (PMC10866534; doi:10.1371/journal.ppat.1011981)
Supplement: Attachment — Submitted filename: Response to Reviewers.docx [file ppat.1011981.s011.docx]

**Part I – Summary**

Reviewer #1: The authors investigated the mechanism of tumor and avian cell apoptosis induced by a virulent NDV infection. The authors showed that NDV infection can reduce lysosome acidification by increasing LMP, resulting of CTSB and CTSD being released into the cytosol, generating ROS and induce mitochondria dysfunction, which leads to mitochondria-dependent apoptosis. The authors also showed the expression of only the HN protein of NDV deglycosylated LAMP1 and LAMP2 via its sialidase activity and therefore destabilized both proteins (reduced protein expression), which could lead to increased LMP. By IP and IF, HN appeared to bind and co-localize with LAMP1 and LAMP2. This is an interesting study adding value to understand the pathogenesis of NDV. However, the following comments should be sufficiently addressed.

Reviewer #2: In the manuscript “The HN protein of Newcastle disease virus induces cell apoptosis through the induction of lysosomal membrane permeabilization” Yu Chen and colleagues elucidate how the Newcastle disease virus induces apoptosis via lysosomal damage. The authors describe how the viral Hemagglutinin neuraminidase (HN) protein digests sialic acids at the end of glycan chains of lysosome-associated membrane protein 1 (LAMP1) and LAMP2, making them vulnerable to degradation and triggering lysosomal membrane permeabilization (LMP). Furthermore, they show how the apoptotic cascades activated by leakage of lysosomal proteins cathepsin B and D end up inducing mitochondria-dependent apoptosis.
This study is nicely constructed, elegantly telling a cohesive story, with extensive amounts of data and very thorough. However, some necessary controls, references and explanations are missing. These concerns need to be addressed to make this a high-quality publication.

**Part II – Major Issues: Key Experiments Required for Acceptance**

Reviewer #1: 1. What happens in cells that is less susceptible to NDV infection? Such as cells that can only support a single round of virus replication? Did the authors observe increased LMP?

**Response:**

Dear reviewer, thank you for your valuable insights. Unfortunately, we did not identify cells supporting a single round of Newcastle disease virus (NDV) replication. Nevertheless, our focus was on elucidating the relationship between NDV-induced lysosomal membrane permeabilization (LMP) and its replicative efficiency, considering two key aspects.

In a separate study, we observed that the genotype VII NDV strain I4 exhibited significantly lower infectivity in various tumor cells compared to the genotype IV NDV strain Herts/33 (Fig 1A). Intriguingly, their HN protein neuraminidase activities were comparable (Fig 1B) (This study, currently under review at PLOS Pathogens, has undergone the initial round of revision, PPATHOGENS-D-23-00910R2). Focusing on this pair of model viruses, we assessed their induction of cellular LMP and the expression levels of LAMP1 and LAMP2 proteins in HeLa cells. Under identical infection dosage (1 MOI) and equivalent time points (18 hpi and 36 hpi), the I4-infected group showed significantly lower LMP levels than the Herts/33-infected group (Fig 1C). Correspondingly, the I4-infected group exhibited notably lower levels of deglycosylation and degradation of LAMP1 and LAMP2 proteins, consistent with the significantly lower expression of the HN protein in the I4-infected group (Fig 1D). However, transfection experiments revealed that the HN protein from I4 induced similar levels of LMP (Fig 1E), as well as deglycosylation and degradation of LAMP1 and LAMP2 proteins (Fig 1F), with essentially identical HN protein expression levels (Fig 1F).

We also examined LMP induced by UV-inactivated NDV. Although ultraviolet (UV) radiation primarily targets nucleic acids, its impact on the biological activity of viral proteins, including the sialidase activity of the HN protein, is relatively minor. Nevertheless, it is noteworthy that both low-dose (0.1 MOI) and high-dose (10 MOI) UV-inactivated NDV failed to induce LMP (Fig 1G) or deglycosylation and degradation of LAMP1 and LAMP2 proteins (Fig 1H) in HeLa cells. Additionally, through a Western blotting assay, we could not detect any intracellular HN protein (Fig 1H). Our hypothesis is that although the UV-inactivated virus membrane retains HN proteins with sialidase activity, NDV primarily releases its nucleocapsid components into host cells through direct membrane fusion mediated by its F protein ^[1]^. Consequently, HN proteins on the virus membrane surface do not enter host cells, resulting in the absence of HN protein in host cells. This might explain why UV-inactivated NDV, despite retaining its HN protein sialidase activity, lacks the ability to induce cellular LMP.

In conclusion, our experimental results suggest a close association between NDV's ability to induce LMP and its replicative capacity, given that replication is a prerequisite for NDV to generate intracellular HN protein. As these results were obtained after concluding the manuscript, we did not find an appropriate place to seamlessly integrate them. Thus, they have not been included in the revised manuscript.

**Fig 1. The correlation between NDV-induced LMP and its replicative capacity (data not shown in revised manuscript).** (A) HeLa cells were infected with the I4 and Herts/33 strains at 0.1 MOI. Cellular supernatants collected at indicated time points post-infection were quantified for viral load based on TCID_50_. (B) HeLa cells were transfected with plasmids expressing HN protein of I4 or Herts/33 at 0.5 µg for 48h, and sialidase activity was measured using the Neuraminidase Assay Kit. (C&D) HeLa cells infected with I4 and Herts/33 at 0.01 MOI for the indicated time points. LMP levels were observed by confocal microscopy (C), and indicated protein levels were detected by Western blotting assay (D). (E&F) HeLa cells were transfected with different doses of indicated plasmids. The LMP levels were observed by confocal microscopy (E), and indicated protein levels were detected by Western blotting assay (F). (G&H) HeLa cells infected with UV-inactivated Herts/33 strain at different MOIs. LMP levels at the indicated time points were observed by confocal microscopy (G), and indicated protein levels were detected by Western blotting assay (H). Manders’ Colocalization Coefficients of galectin 3 with LAMP1 were quantified by ImageJ software and shown on the right of (C&E&G). Scale bars, 20 µm. Error bars represent SDs for a triplicate analysis of three independent experiments (A&B), or SDs for 15 cells (C&E&G). Significance was assessed using Two-way ANOVA with Dunnett's multiple comparisons test (A) or One-way ANOVA with Dunnett's multiple comparisons test (B&C&E&G). NS means no significant difference, *****P*< 0.0001.

2. Does lentogenic NDV also has the same effects? It was previously reported that the HN protein of NDV determines tropism of virulency (PMID: 15047833). The HN protein of the Lentogenic strains such as La sota seems to have less NA activity as compared to virulent strain BC. The authors should compare avirulent strain and virulent strain by either infection or transfection to see if this is universal to all NDV HN proteins.

**Response:**

Dear reviewer, thank you for your valuable insights. To explore the universality of HN protein-induced LMP across diverse NDV strains, we constructed plasmids expressing the HN protein using cDNA from six distinct NDV strains. These strains included lentogenic strains La Sota (GenBank Accession Number: AF077761) and LX (KF494201), mesogenic strains Mukteswar (EF201805.1) and JS/07/04/Pi (FJ766530), and virulent strains F48E8 (FJ436302.1) and Kuwait256 (MK978147). Sialidase activity assays revealed significantly higher activity in the HN protein derived from virulent and mesogenic strains compared to lentogenic strains (S7A Fig). Furthermore, Western blotting and confocal microscopy experiments demonstrated that transfection of HeLa cells with expression plasmids from different strains induced deglycosylation and degradation of LAMP1 and LAMP2 (S7B Fig), along with LMP (S7C Fig). Remarkably, the induction levels were more pronounced for HN proteins from virulent and mesogenic strains (S7B & S7C Fig). These findings suggest that the induction of LMP, mediated by the HN protein, demonstrates a degree of universality across distinct NDV strains and is positively correlated with the sialidase activity of the HN protein. In addition, this suggests that the sialidase activity of the HN protein may have a potential regulatory role in the virulence of NDV by inducing LMP. We have added relevant content to the Results (line 331-343) and Discussion (451-455) sections in the revised manuscript

3. Figure 3A and 3E, do CTSB and CTSD inhibitors or CTSB and CTSD knockdown reduce NDV infectivity? Please show the expression levels of viral protein(s), such as NP.

**Response:**

Dear reviewer, thank you for your valuable insights. Initially, we did not consider the impact of CTSB and CTSD inhibitors, as well as siRNAs, on NDV infectivity. Following the previous experimental protocol (Fig 2A&2D, data showed in below), both the inhibitors and siRNAs exhibited a certain degree of inhibition on NDV replication (Fig 2B&2C&2E, data showed in below). These results are consistent with the outcomes presented in Figure S3, confirming that the inhibition of LMP suppresses NDV replication.

To eliminate the potential influence of CTSB and CTSD inhibitors and siRNAs on NDV infectivity, we adjusted the experimental design. Following the modified experimental design shown in Fig 3A&H, we observed that the inhibitors and siRNAs of CTSB and CTSD significantly suppressed NDV-induced cell apoptosis (Fig 3B&3E) or the expression of apoptosis-related proteins (Fig 3I) in the absence of a significant impact on the viral NP protein (Fig 3D&3G&3I). Based on this, we propose that LMP promotes NDV-induced apoptosis through the involvement of CTSB and CTSD. Due to modifications in experimental protocol, we re-carried out the related experiments involving CTSB and CTSD inhibitors and siRNAs according to the modified protocol. The updated results are shown in Fig 4A, 4B, 4H, 4K, and Fig 5B in the revised manuscript.

**Fig 2 (data not shown in revised manuscript).** (A) HeLa or DF-1 cells were pretreated with Ca-074 (20 µM), Pep A (20 µM), DMSO, or mock-treated for 3h. Then the cells were infected with Herts/33 at 0.01 MOI for 24h in the culture medium containing corresponding chemicals. The NP protein levels in HeLa (B) and DF-1 (C) cells were detected by Western blotting assay. (D) HeLa cells were transfected with si-CTSB, si-CTSD, si-NC or mock-transfected for 48h. Then, the cells were infected with Herts/33 at 0.01 MOI for 24h. The NP protein level in HeLa was detected by Western blotting assay (E). GAPDH was used as a normalized control.

4. Figure 3E and 3Q, the authors should show protein expression in mock infected cells with knockdown of CTSB and CTSD as a control.

**Response:**

Dear reviewer, thank you for your valuable insights. In response to your suggestion, we repeated the relevant experiments, incorporating a mock-infected group with knockdown of CTSB and CTSD as a control. The updated results are now presented in Fig 3I and Fig 4H in the revised manuscript.

5. The phenotype in figure 1-4 was very clear when infecting cells with NDV at MOI of 0.01 for 24 hours. However, the reduction of LAMP1 expression and deglycosylation in figure 5C was only significant when infecting cells at MOI of 1 for 24 hours. On the other hand, LAMP2 seems to be more affected by NDV infection at MOI of 0.01 than LAMP1. But in Figure 5F, after 12 hours of infection at MOI of 0.01, LAMP1 is already deglycosylated and degraded? And F5I is 6 hours after infection at MOI of 0.01? Please clarify.

**Response:**

Dear reviewer, thank you sincerely for your valuable suggestions. Upon a meticulous review of the relevant experimental records, we have identified an oversight in the plotting process. Regrettably, we incorrectly labeled the viral infection doses in Fig 6C, 6D, and 6E. Despite specifying 0.01 MOI in the Figure Legends, the figures mistakenly indicate 1 MOI. Additionally, the different infection doses on the right should commence from 0.001 MOI. We appreciate your understanding and have promptly corrected this error in the revised figures. Upon correction, we noticed that the levels of deglycosylation and degradation of LAMP1 and LAMP2 proteins still exhibit differences between Fig 6C, Fig 6F, and Fig 2A. Specifically, in Fig 6C, significant deglycosylation and degradation are observed only after 24h of 0.01 MOI infection, while in Fig 6F and Fig 2A, noticeable deglycosylation and degradation are observed after 12h of 0.01 MOI infection. Considering the potential errors in the experimental process for Fig 6C (virus dilution, protein loading in the Western blot, etc.), we have repeated the experiment in Fig 6C and have submitted new experimental results.

And F5I is 6 hours after infection at MOI of 0.01? In Fig 6I, HeLa cells were initially infected with Herts/33 at 0.01 MOI. After 6h of viral infection, we collected a sample as a control (first lane), while the remaining cells were co-treated with CHX and various drugs or mock-treated for an additional 12h (lanes 2-7). Therefore, in Fig 6I, the first lane represents the results after 6h of viral infection, while the remaining lanes depict outcomes after 18h of viral infection.

Reviewer #2: 1. Ju and colleagues showed 2015 in JVI something very similar for the Neuraminidase of Influenza A virus. This paper needs to be referenced.

**Response:**

Dear reviewer, thank you sincerely for your valuable suggestions. Following your advice, we have referenced this article in the revised manuscript and briefly discussed the differences in inducing LMP between IAV and NDV in the Discussion section (456-467).

2. Figure 3E: a non-target siRNA control is needed (similar to how it is done in Figure 6K)

**Response:**

Dear reviewer, we're grateful for your valuable input. In response, we've repeated relevant experiments and integrated the latest results into the revised manuscript (Fig 3I).

3. Figure 5I and the corresponding result section need to address some inconsistencies:
a. Figure 5F and I: In Figure 5F, NDV 12hpi and CHX treatment, LAMP1 and LAMP2 are fully degraded. In Figure 5I, LAMP1 and LAMP2 have strong bands 12h post CHX treatment and NDV infection (mock other treatments). Why the difference in expression between the experiments?

**Response:**

Dear reviewer, Thank you for your valuable inquiry. In Figure 6F, we initially infected HeLa cells with 0.01 MOI NDV. After 12h of viral infection, we added CHX to the cell culture medium. Subsequently, we monitored the remaining levels of LAMP1 and LAMP2 proteins in each group every 3h (Refer to Fig. 3A below for the schematic diagram). In Figure 6I, we first infected HeLa cells with 0.01 MOI NDV. Following 6h of viral infection, we added CHX and other drug inhibitors to the cell culture medium. After 12h of drug treatment, we assessed the levels of LAMP1 and LAMP2 proteins in each cell group (Refer to Fig. 3B below for the schematic diagram.). Due to the disparate objectives and durations of drug treatments in these two experiments, the corresponding viral infection times also differ. To avoid any potential misunderstanding, we have provided detailed explanations of the specific steps in the Figure legend of Fig. 6 (line 810-828).

**Fig 3 Schematic diagram of experimental design for Fig 6F (A) and Fig 6I (B). Data not shown in the revised manuscript.**

b. Figure 5I: All the bands of samples treated with CHX appear to be lower than samples not treated with CHX (h0). Does CHX lead to deglycosylation? Please add possible explanation

**Response:**

Dear reviewer, thank you for your valuable inquiry. As an inhibitor of eukaryotic protein translation, CHX, to our knowledge, has not been reported to possess deglycosylation capabilities. Moreover, as illustrated in Figure 6F, CHX treatment does not impact the glycosylation of LAMP1 and LAMP2 in the Mock group. We attribute the observed deglycosylation phenomenon to the effects of NDV infection. In the experiment presented in Figure 6I, protein samples from the CHX (0h) group originate from HeLa cells infected with NDV for 6h, while the remaining protein samples are derived from HeLa cells subjected to continued treatment with CHX and other drugs in the presence of NDV for an additional 12h. In other words, the actual viral infection time for the CHX combination treatment group is 18h, representing a 12h extension compared to the CHX (0h) group. This extension is likely the cause of the deglycosylation observed in LAMP1 and LAMP2.

c. Figure 5I: PepA, 3-MA and MG132 appear to increase degradation of LAMP1 and LAMP2 compared to mock treated cells. Please address

**Response:**

Dear reviewer, thank you for your valuable suggestions. Indeed, as shown in the previous Fig 5I, PepA, 3-MA, and MG132 appeared to enhance the degradation of LAMP1 and LAMP2 compared to mock-treated cells. However, the statistical analysis indicates no significant differences between these groups. This inconsistency may be attributed to the choice of representative images, as in the remaining two experiments, there were no visible differences between these groups. To avoid potential misunderstandings, we have replaced the previous Figure 5I with the revised Figure 6I in the manuscript. Below are the results from three independent Western blot (WB) experiments.

**Part III – Minor Issues: Editorial and Data Presentation Modifications**

Reviewer #1: -The role of torin 1 in figure 1A should be mentioned in the result

**Response:**

Dear reviewer, thank you for your valuable suggestions. We have supplemented the relevant description in the revised manuscript (line 104-105).

-To mutate 10 conserved amino acids in HN neuraminidase active sites, why not do alanine scanning for all 10 residues? For example, I175L doesn’t seem to be a big change.

**Response:**

Dear reviewer, we're grateful for your valuable input. In this experiment, we mainly followed the methodology outlined by Connaris, H et al. ^[2]^. In their research, they introduced mutations at the ten conserved sites, altering them to different amino acids, and investigated the impact of these mutations on the sialidase activity of the HN protein. Based on their findings, we selected the ten mutation patterns that demonstrated a significant effect on sialidase activity. These patterns include R174A, I175E, E258D, E299A, E401D, R416A, R498A, E547D, Y299A, and Y317F. However, during the construction of the I175E mutation plasmid, we consistently encountered unintended mutations at other amino acid positions. Consequently, we opted to use I175L as a substitute for I175E. Thus, the final set of ten mutations includes: R174A, I175L, E258D, E299A, E401D, R416A, R498A, E547D, Y299A, and Y317F.

Reviewer #2: 1. Some bar graphs show data points, some do not. Preferably, show data points for all bar graphs.

**Response:**

Dear reviewer, thank you for your kind advise. We have revised all the figures and included data points for all bar graphs.

2. Define abbreviations: line 169 si-CTSB；Line 372 Ad5 and DENV MOI

**Response:**

Dear reviewer, thank you for your valuable suggestions. We have provided definitions for si-CTSB (line 171-172) and MOI (line 569). However, as Ad5 and DENV have already been defined in the Introduction (line 76-77), we did not repeat the definitions in the Discussion.

3. The authors should briefly discuss why LAMP3 stays stable upon NDV infection whereas LAMP1 and LAMP2 are being degraded (as depicted in Figure 2A). What makes LAMP3 different?

**Response:**

Dear reviewer, we appreciate your valuable suggestions. Concerning the specific degradation of LAMP1 and LAMP2 during NDV infection, with no impact on LAMP3, LIMP II, and LAPTM5, the exact reasons remain elusive. However, considering the structural and functional variations among these proteins, two factors are hypothesized to contribute to the observed differences in protein stability.

Firstly, LAMP1 and LAMP2, characterized by single-pass transmembrane structures with similar lengths and 37% amino acid sequence homology, contrast with LAMP3 (four-pass transmembrane), LIMP II (two-pass transmembrane), and LAPTM5 (five-pass transmembrane) ^[3-5]^. The more intricate structures of LAMP3, LIMP II, and LAPTM5 may impact their interaction with the HN protein.

Secondly, LAMP1 and LAMP2, representing nearly half of lysosomal membrane proteins, while LAMP3, LIMP II, and LAPTM5 show comparatively lower expression on lysosomal membranes ^[4, 6]^. Additionally, apart from LAPTM5, the glycosylation pattern differs: LAMP1 and LAMP2 possess 24 glycosylation sites, while LAMP3 has 7, and LIMP II has 10 ^[4]^. The abundant expression and multiple glycosylation sites of LAMP1 and LAMP2 likely offer ample "sites" for the HN protein to mediate deglycosylation.

In general, the unique structural, wide distribution and glycosylation features of LAMP1 and LAMP2, compared to LAMP3, LIMP II, and LAPTM5, may contribute to the NDV-induced degradation. However, further investigations are essential to unveil the precise underlying mechanisms.

4. Figure 2C and 2F: It appears that the amount of cytoplasmic cathepsin B decreases 36hpi compared to 24hpi while the activity of cytoplasmic cathepsin B increased in the same time span. Possible explanation?

**Response:**

Dear reviewer, thank you very much for your valuable question. Indeed, as shown in Figure 2C and Figure 2F, the trends in cytoplasmic cathepsin B based on Western Blot (WB) detection and fluorescence intensity detection are inconsistent at the 24h and 36h time points. We observed this issue during the experiment. However, through a detailed analysis of the raw experimental results, we confirmed the reliability of these findings.

Cytoplasmic Cathepsin B is derived from the inactive proenzyme (prepro-CTSB), and its maturation pathway in the cell is briefly as follows: prepro-CTSB is synthesized in the ribosome and then transported to the endoplasmic reticulum, where it undergoes hydrolysis to form the proenzyme (pro-CTSB) containing the propeptide and catalytic domain. Subsequently, it is transported to the intracellular lysosome, where it undergoes automatic hydrolysis under acidic conditions, removing the propeptide and generating the active mature CTSB.

Based on the maturation process of CTSB, we analyzed the potential reasons for this inconsistency, which may be related to the specificity of different detection methods. Figure 2C presents the results of the Western Blot experiment, detecting the total Cathepsin B in the cell, including prepro-CTSB, pro-CTSB, and mature CTSB. On the other hand, Figure 2F measures the concentration of CTSB with proteolytic activity in the cell, mainly representing mature CTSB. This is likely the primary reason for the observed differences in results between the two detection methods. We have added the above possible explanation in the revised manuscript (line 423-438).

5. Figure 3 is very busy. Maybe split figure into two or move some of the data to supplementary figures.

**Response:**

Dear reviewer, thank you very much for your valuable suggestion. For reader comprehension, we have divided Figure 3 into two parts in the revised manuscript (Fig 3 and Fig 4).

6. Figure 3T: Labelling Mitochondrial fraction appears doubled; Figure 3O, P: The NS are shifted

**Response:**

Dear reviewer, thank you for your valuable suggestion. We have resolved the labeling issues, as depicted in the revised manuscript's Fig 4F, 4G, and 4K.

7. Figure 5E: LAPTM5 is decreasing in the 24hpi column compared to the 1 MOI column?

**Response:**

Dear reviewer, thank you for valuable suggestion. Indeed, except for LAPTM5, the gray values of LAMP3 and LIMPII in 24hpi column are slightly lower than those in 0.01 MOI column. As the samples for 24hpi column and 0.01 MOI column are from two separate experiments. A certain level of protein degradation may have occurred in the 24hpi column samples, resulting in slightly lower gray values for LAMP3, LIMPII, and LAPTM5 compared to 0.01 MOI column. To mitigate the error arising from protein degradation in different batches, we prepared all samples simultaneously and re-conducted the Western blotting assay. The results indicated that regardless of 24hpi column and 0.01 MOI column, there was no significant impact on the expression of LAMP3, LIMPII, and LAPTM5 proteins (Fig 6E). Moreover, there was no notable difference in the expression levels of these three proteins between the24hpi column and t0.01 MOI column (Fig 6E). We have resubmitted new experimental results (Fig 6E) in the revised manuscript.

8. Figure 6A and B: In Figure 6A HN leads to deglycosylation, in Figure 6B HN leads to deglycosylation AND degradation. Where does the difference come from? Transfected with different amounts of plasmid?

**Response:**

Dear reviewer, thank you for your valuable suggestion. In Figure 6A, the transfection amount of the HN protein expression plasmid is 0.5 µg. Through gray analysis, in both Figure 6A and Figure 6B, transfection with the HN protein expression plasmid can induce deglycosylation and degradation of LAMP1 and LAMP2 protein. However, in Figure 6B, this degradation appears to be more pronounced, possibly because the exposure time in Figure 6B is shorter, making the differences in grayscale values between different lanes appear more obvious. To prevent any misunderstanding, we have repeated the experiments for Figure 6B and supplemented new Western Blot images in the revised manuscript (Fig 7B).

9. Figure 6E and F are too small

**Response:**

Dear reviewer, thank you very much for your valuable suggestions. In the revised manuscript, we have reformatted the original Figure 6, and the results are presented in Figure 7.

10. The labelling of western blots in supplementary figure 5 is inconsistent with the other figures, using anti-LAMP1/LAMP2/Flag and so forth instead of just LAMP1 etc.

**Response:**

Dear reviewer, thank you very much for your valuable advice. In the revised manuscript, we have made modifications to the labeling of all Western Blotting images to enhance clarity and readability.

11. Pepstatin A is an aspartic acid protease inhibitor, not a cathepsin D specific inhibitor (line 160).

**Response:**

Dear reviewer, thank you very much for your valuable suggestion. We have revised the wording in question in the revised manuscript according to your advice (line 163).

**Reference:**

[1]	Ganar K, Das M, Sinha S, Kumar S. Newcastle disease virus: current status and our understanding [J]. Virus Res, 2014, 184(12): 71-81.

[2]	Connaris H, Takimoto T, Russell R, Crennell S, Moustafa I, Portner A, Taylor G. Probing the sialic acid binding site of the hemagglutinin-neuraminidase of Newcastle disease virus: identification of key amino acids involved in cell binding, catalysis, and fusion [J]. J Virol, 2002, 76(4): 1816-1824.

[3]	Wilke S, Krausze J, Büssow K. Crystal structure of the conserved domain of the DC lysosomal associated membrane protein: implications for the lysosomal glycocalyx [J]. BMC Biol, 2012, 10(19): 62.

[4]	Alessandrini F, Pezzè L, Ciribilli Y. LAMPs: Shedding light on cancer biology [J]. Semin Oncol, 2017, 44(4): 239-253.

[5]	Eskelinen E L. Roles of LAMP-1 and LAMP-2 in lysosome biogenesis and autophagy [J]. Mol Aspects Med, 2006, 27(5-6): 495-502.

[6]	Ueo A, Kubota M, Shirogane Y, Ohno S, Hashiguchi T, Yanagi Y. Lysosome-Associated Membrane Proteins Support the Furin-Mediated Processing of the Mumps Virus Fusion Protein [J]. J Virol, 2020, 94(12): e00050-00020.
