# Supplementary material for: The HN protein of Newcastle disease virus induces cell apoptosis through the induction of lysosomal membrane permeabilization
Source: PLoS Pathog. 2024 Feb 14;20(2):e1011981. doi: 10.1371/journal.ppat.1011981 (PMC10866534; doi:10.1371/journal.ppat.1011981)
Supplement: Attachment — Submitted filename: Response to Reviewers.docx [file ppat.1011981.s012.docx]

**Reviewer's Responses to Questions**

**Part I - Summary
Please use this section to discuss strengths/weaknesses of study, novelty/significance, general execution and scholarship.**

**Reviewer #1: The authors addressed most of my comments sufficiently**

**Reviewer #2: The authors have addressed my concerns and revised the manuscript accordingly.**

**Part II – Major Issues: Key Experiments Required for Acceptance
Please use this section to detail the key new experiments or modifications of existing experiments that should be absolutely required to validate study conclusions.

Generally, there should be no more than 3 such required experiments or major modifications for a "Major Revision" recommendation. If more than 3 experiments are necessary to validate the study conclusions, then you are encouraged to recommend "Reject".**

**Reviewer #1: no major issues**

**Reviewer #2: (No Response)**

**Part III – Minor Issues: Editorial and Data Presentation Modifications
Please use this section for editorial suggestions as well as relatively minor modifications of existing data that would enhance clarity.**

**Reviewer #1: please clarify what is si-NC in the method section since this control was newly added**

**Response：**Dear reviewer, thanks for your valuable insights. Following your advice, we have added relevant descriptions about si-NC in the methods section (line 562-563).

**Reviewer #2: (No Response)**
